# ‘We are the bridge’: an implementation research study of SEWA Shakti Kendras to improve community engagement in publicly funded health insurance in Gujarat, India

**DOI:** 10.1136/bmjgh-2022-008888

**Published:** 2022-09-23

**Authors:** Susan Thomas, Sharmada Sivaram, Zubin Shroff, Ajay Mahal, Sapna Desai

**Affiliations:** 1 Lok Swasthya SEWA Trust, Self-Employed Women’s Association (SEWA), Ahmedabad, Gujarat, India; 2 Population Council Consulting, New Delhi, India; 3 Alliance For Health Policy and System Research, Geneva, Switzerland; 4 The University of Melbourne Nossal Institute for Global Health, Carlton, Victoria, Australia; 5 Population Council Institute, New Delhi, India

**Keywords:** Health insurance, Health systems evaluation

## Abstract

**Introduction:**

India’s efforts towards universal health coverage include a national health insurance scheme that aims to protect the most vulnerable from catastrophic health expenditure. However, emerging evidence on publicly funded health insurance, as well as experience from community-based schemes, indicates that women face specific barriers to access and utilisation. Community engagement interventions have been shown to improve equitable utilisation of public health services, but there is limited research specific to health insurance. We examined how existing community-based resource centres implemented by a women’s organisation could improve women’s access to, and utilisation of, health insurance.

**Methods:**

We conducted an implementation research study in Gujarat, India to examine how SEWA Shakti Kendras, established by the Self-Employed Women’s Association, worked to improve community engagement in health insurance. SEWA organises women in the informal sector and provides social protection through health, insurance and childcare services. We examined administrative data, programme reports and conducted 30 in-depth qualitative interviews with users and staff. Data were analysed thematically to examine intervention content, context, and implementation processes and to identify enablers and barriers to improving women’s access to health insurance through SEWA’s community engagement approach.

**Results:**

The centres worked through multiple channels—doorstep services, centre-based support and health system navigation—to strengthen women’s capability to access health insurance. Each centre’s approach varied by contextual factors, such as women’s digital literacy levels and rural–urban settings. Effective community engagement required local leadership, strong government partnerships and the flexibility to address a range of public services, with implementation by trusted local health workers.

**Conclusion:**

SEWA Shakti Kendras demonstrate how a local, flexible and community-based model can serve as a bridge to improve utilisation of health insurance, by engaging women and their households through multiple channels. Scaling up this approach will require investing in partnerships with community-based organisations as part of strategies towards universal health coverage.

**WHAT IS ALREADY KNOWN ON THIS TOPIC**

- Engaging citizens in the design and delivery of public services can improve reach, quality and equity.
- Current evidence on community engagement focuses on health service delivery, with limited evidence specific to health insurance schemes.

**WHAT THIS STUDY ADDS**

- Community resource centres that engage with women, their families and the health system through multiple channels can support utilisation of health insurance.
- Local leadership, flexibility, trust and government partnerships are key to effective implementation.

**HOW THIS STUDY MIGHT AFFECT RESEARCH, PRACTICE OR POLICY**

- Trusted community-based organisations can serve as a link between publicly funded health insurance and community members, particularly women.
- Approaches to improve outreach for health insurance can include doorstep information provision, health system navigation and one-stop services at community resource centres.
- Government partnerships with community-based organisations can play an important role in improving equitable utilisation of health insurance schemes.

## Background

There is widespread recognition that engaging people to improve the reach, quality and equity of public health services is critical to achieving universal health coverage (UHC).^1–3^ Evidence on community engagement in health, while extensive, focuses on health services, with limited research specific to health insurance. Recent community engagement efforts to improve equitable utilisation of health insurance—an increasingly important component of UHC strategies in low-income and middle-income settings—include community-based awareness-raising, brokers and staff to provide navigation support and formal grievance redressal mechanisms.^4 5^

In India, the potential of community engagement initiatives is particularly relevant in the backdrop of the flagship ‘Ayushman Bharat’ scheme introduced in 2018. The scheme has two pillars: Health and Wellness Centres (HWCs) to strengthen primary healthcare and the Prime Minister’s Jan Arogya Yojana (PMJAY), a national, publicly funded health insurance scheme that covers 40% of the population (approximately 500 million people), based on income and vulnerability criteria.^6^ The insurance offers cashless services in empanelled hospitals for inpatient care and selected outpatient packages. In response to barriers to enrolment and utilisation identified in earlier insurance schemes,^7^ PMJAY introduced several adaptations. It reframed the scheme as entitlement-based rather than requiring enrolment, wherein eligible individuals can access benefits directly at the point of service by showing proof of identification or eligibility. Beneficiaries can obtain physical PMJAY cards through common service centres and empanelled hospitals, which was intended to help patients avoid document processing at the time of treatment. Dedicated staff—*Arogya Mitras*—are available within empanelled hospitals to support beneficiaries during admission; their role does not include community engagement. In India’s federal structure, the centrally-funded PMJAY complements or expands coverage provided by previously established state-level health insurance schemes.

Early studies on PMJAY indicate increased coverage of cards, with large variations in hospital empanelment and service utilisation across states. Studies report mixed results regarding financial protection and out-of-pocket health expenditure.^8–11^ While the scheme continues to improve through innovations in technology, gender-specific barriers pose a persistent challenge. Analyses of both PMJAY and state-level schemes report lower utilisation amongst women compared with men. A 2020 analysis of gender differentials in PMJAY utilisation found that, despite similar insurance card coverage, women comprised a slightly lower proportion of all claims submitted compared to men (48.5% vs 51.4%).^12^ Excluding obstetric and gynaecological procedures, which were mostly delivery care, women comprised only 43% of all claim authorisations. This proportion remains the same amongst older women past childbearing age (45–64 years). In Andhra Pradesh’s Arogyasri scheme, women comprised 42% of all non-obsetetric or gynaeocogical hospital admissions and 39% of all treatment costs--lower than men across all age groups.^13^ In Rajasthan, women comprised a lower share of claims (43%) and hospital visits (45%) than men, leading the authors to conclude that the state health insurance programme’s ‘spending is male-skewed’.^14^

Women’s participation in caregiving roles and other forms of unpaid work, mobility constraints and limited control over household resources are well-known barriers to health service use. Specific to health insurance, gender inequities are related to: how women’s biological risk factors, experience of illness and social contexts differ from men; women’s limited decision-making power regarding use of health services and insurance; and the specific vulnerabilities of women workers in the informal economy and lower rungs of the formal sector.^15^ Further, women’s lower literacy and access to technology have resulted in lower awareness of scheme benefits and processes.^15–19^ A 2020 analysis of Tamil Nadu’s state health insurance scheme highlighted gendered barriers at multiple levels—household, community, state and market—to explain women’s lower utilisation of the scheme.^20^ The study highlighted the need for greater community engagement in the design, implementation or monitoring of the scheme. In-depth research on women’s use of insurance in Chhattisgarh pointed to women’s higher vulnerability to incurring out-of-pocket expenditure despite being insured, harassment due to inability to pay and greater likelihood of undergoing unnecessary procedures.^21^

In light of these gender inequities, we aimed to explore how a community engagement initiative by the Self-Employed Women’s Association (SEWA), an organisation of women workers in India’s informal economy, could improve women’s use of health insurance. SEWA initiated community-based resource centres, SEWA Shakti Kendras (SSK), to work in partnership with government to improve women’s access to entitlements such as pensions, health services and more recently, health insurance. As part of a multicountry initiative,^22^ we conducted an implementation research study to explore the content, context and processes through which interventions by SSKs engaged with women to improve access to health insurance along with other services.

## Methods

### Setting

SEWA, founded in 1972 in Gujarat, has approximately 1.8 million women worker members across 18 states, in four main occupation categories: vendors and hawkers, home-based workers, labour or service providers and rural producers. Members are among the poorest and most vulnerable of India’s workers in the informal sector, characterised by low wages and limited social security benefits.^23^ SEWA’s health organisation Lok Swasthya SEWA Trust (LSST) provides health services to SEWA members, their families and communities while organising women to access healthcare entitlements.^24^ LSST community health workers, who are SEWA members themselves from the same communities, are known as *aarogya sevikas* (*sevikas*). They provide basic, first-level community contact care such as treatment and screening for common illnesses, with a focus on health awareness, community mobilisation and health system navigation support.

LSST initiated SSKs in Gujarat in 2015 as an extension of its longstanding community health programme. SSKs are community-based resource centres that work to improve women’s access to government benefits through information provision and navigation support. Implemented by *sevikas* and other local leaders, they conduct home visits, organise community meetings and form government linkages to ensure last mile delivery of public entitlements. These linkage centres fulfil SEWA’s commitment to ensuring social protection for members, including health, childcare and insurance. In response to the introduction of PMJAY in 2018, SSK activities expanded to include publicly funded health insurance.

The study was conducted in rural and urban Ahmedabad, in Gujarat. The PMJAY is implemented alongside the state government *Mukhyamantri Amrutum* (MA) scheme, which covers catastrophic hospitalisation among low-income families. A total of 2,730 hospitals, of which 70% are in the public sector, have been empanelled under PMJAY in Gujarat, with 2,867,866 claims submitted since inception till May 2022.^25^ National survey data indicate that most inpatient admissions (excluding those for childbirth) occur in the private sector, particularly in urban areas (table 1).

**Table 1:** Key indicators for Gujarat

Total population	60 million*	
Rural population	57.4%*	
Sex ratio (females per 1000 males)	965†	
Female literacy rate	73.5%†	
Women who own a mobile phone	48.8%†	
Households with any member covered under any health insurance	39.0%†	
Infant mortality rate (per 1000 births)	31.2†	
Maternal mortality ratio (per 100 000 live births)	75‡	
% rural hospitalisation in public sector	40.1%§	
% urban hospitalisation in public sector	21.3%§	
% institutional delivery in public sector	43.3%†	
High/very high blood sugar level/taking medicine to control blood sugar	15.8%† (women)	16.9%† (men)
Hypertension among adults (elevated blood pressure/taking medicine	20.6%† (women)	20.3%† (men)

*Census of India (2011).

†National Family Health Survey 5 (2019–2021). Health insurance includes public and private insurance.

‡Sample Registration System report (2016–2018).

§National Sample Survey 75th Round (2017–2018). Hospitalisation excludes childbirth.

### Approach

**Figure 1:**
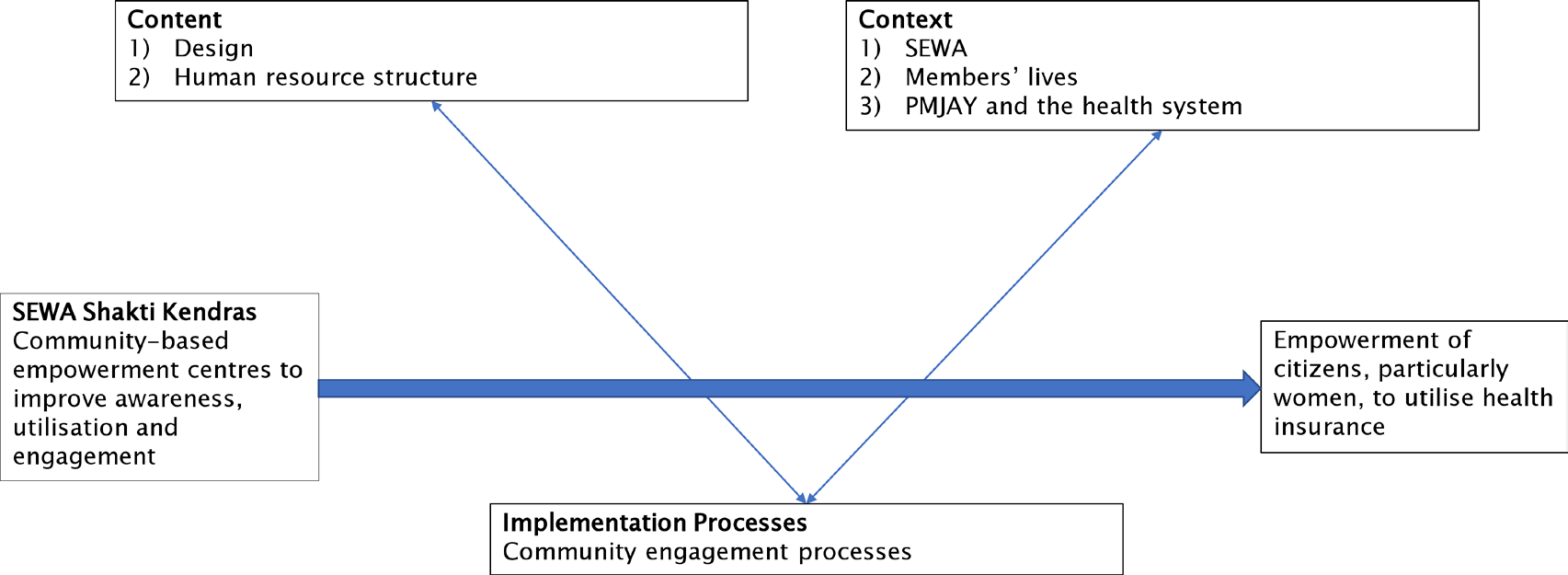
Framework for data collection. PMJAY, Prime Minister’s Jan Arogya Yojana; SEWA, Self-Employed Women’s Association.

This implementation research study was conducted jointly by LSST and the Population Council Institute. We draw from health systems implementation research methods^26^ to examine (1) intervention content (2) context and (3) implementation processes (figure 1). We aimed to identify factors that could inform transferability of the SSK approach to other settings and for potential scale-up by SEWA.

### Data collection

We purposively selected six rural and urban SSKs in Ahmedabad district (online supplemental material) to ensure a geographical spread and a mix of older and newer SSKs. We used secondary sources, including LSST reports, administrative data and previous case studies, and collected primary data with SSK users and the implementing team.

10.1136/bmjgh-2022-008888.supp1 Supplementary data

We conducted a baseline rapid needs assessment in early 2020 among 3,663 PMJAY eligible households on service utilisation in the 6 SSK areas. Using this household survey listing, we randomly selected 22 women for in-depth, semi-structured phone interviews. We conducted in-depth interviews with key LSST staff (n=3) and community health workers (n=6) to examine content and context, explore implementation processes and to better understand enablers and barriers to accessing PMJAY through SSKs. We reviewed reports and secondary data to understand content and processes of the intervention and to identify themes to explore further in interviews.

Due to COVID-19 restrictions in India, initiated in March 2020 and extended through the study period, all interviews were conducted telephonically in Gujarati, with text-message consent forms shared in advance. Interviews were recorded after obtaining consent from respondents. LSST staff not directly involved with SSKs conducted interviews with users. SD interviewed LSST staff and community health workers, building on previous experience working with SEWA. Interviewers’ familiarity with SEWA lent itself to generating rich contextual information, which was particularly important given all interviews were conducted telephonically.

Recordings were translated and transcribed from Gujarati into English and prepared for coding and analysis. After transcription, we conducted a thematic analysis using NVivo R1, with an emphasis on examining intervention content, context and processes to identify enablers and barriers to SSK’s community engagement approach. The research and implementation teams discussed the codebook and interim research findings were discussed iteratively. We removed identifying information for respondents and used pseudonyms in this manuscript.

## Findings

Study findings are organised by intervention content, implementation processes and contextual factors. Intervention content explains the design, specifically the intended goals and human resource structure intended by LSST. We then describe implementation processes related to community engagement as observed through three channels: information provision, documentation support and exposure visits. The final section of the findings describes contextual factors that influenced the programme pathway.

### Intervention content

#### Design

**Figure 2:**
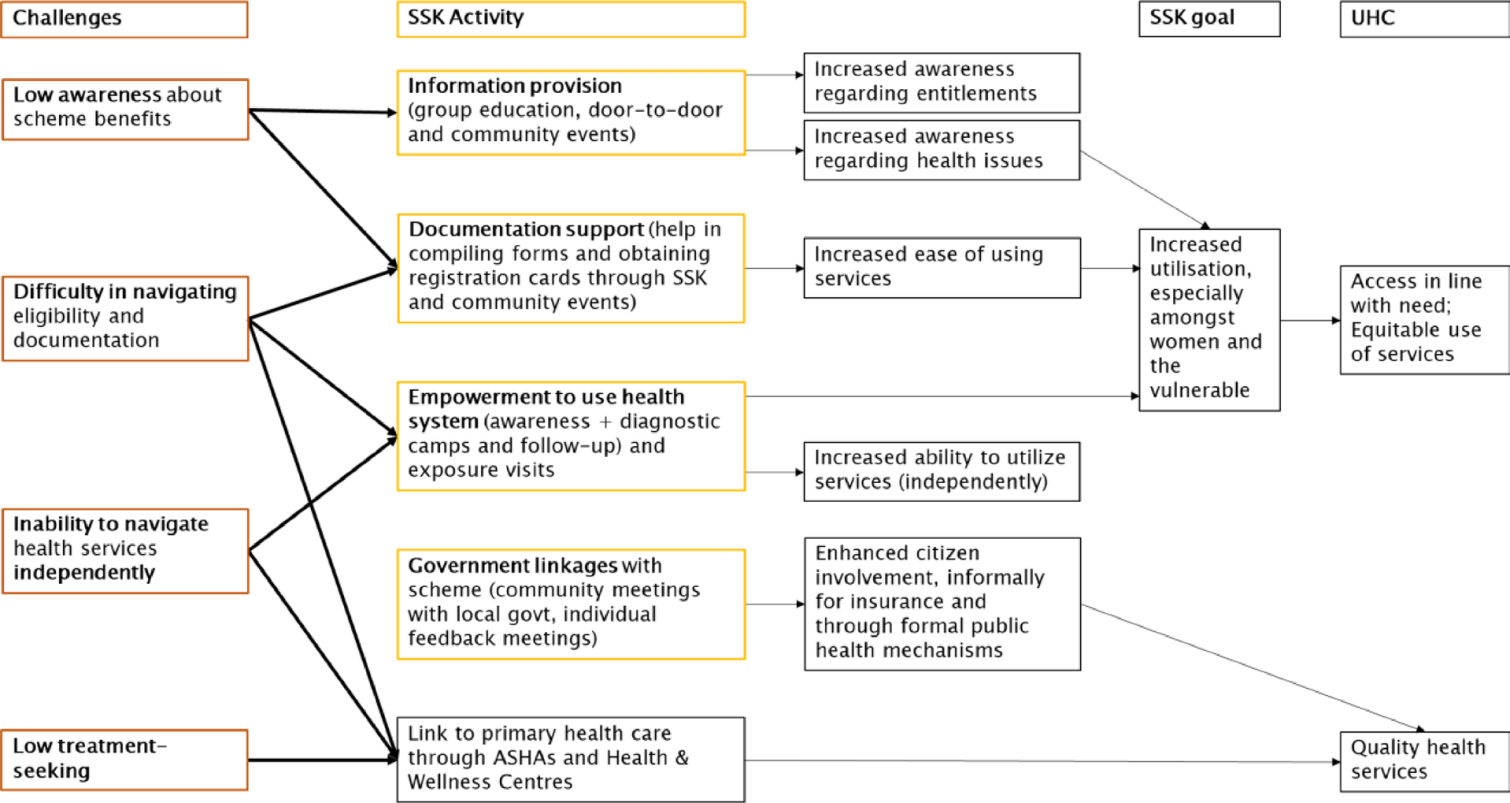
SSK’s intervention pathway. SSK, SEWA Shakti Kendras; VHSNC, village health, sanitation and nutrition committee.

SSKs were envisioned as one-stop centres to serve a range of needs, including access to government benefits, a safe space for women to meet and as a way to bring communities and government together. LSST identified key challenges to women’s utilisation of health insurance: low awareness about scheme benefits; documentation and utilisation processes that were difficult to navigate for low-income/low-literacy women; inability to navigate the health system independently; and low treatment-seeking in general due to social and economic factors. Accordingly, the SSKs focused on a set of community engagement activities that aimed to: increase awareness; support documentation processes and access to cards; strengthen women’s capability to use the health system independently; and strengthen linkages with the scheme itself to share women’s feedback and resolve issues. SSKs also organised community-based mobile health check-up camps to facilitate access to government doctors, diagnostic tests and medicines (figure 2). SSKs aimed to forge strong partnerships with the government through existing community-based mechanisms, such as nationally established village health, sanitation and nutrition committees (VHSNCs) and directly engaging with local government, known as *panchayati raj* institutions. SSKs aimed to address implementation bottlenecks locally, while LSST leadership could address broader policy-level issues through engagement with the district administration or state government.

#### Implementation structure

In urban areas, SSKs cover between 600 and 1000 households and an entire village in rural areas, ranging up to 1500 households. Within each SSK area, approximately 200–500 households are typically eligible for PMJAY. Each SSK is a physical centre, housed in a village community centre or rented space. SSKs operate during hours convenient for informal workers; *sevikas* are typically available by phone at any time. SSKs intended to serve as a meeting place for local government representatives and community members to engage in dialogue, facilitated by LSST.

**Figure 3:**
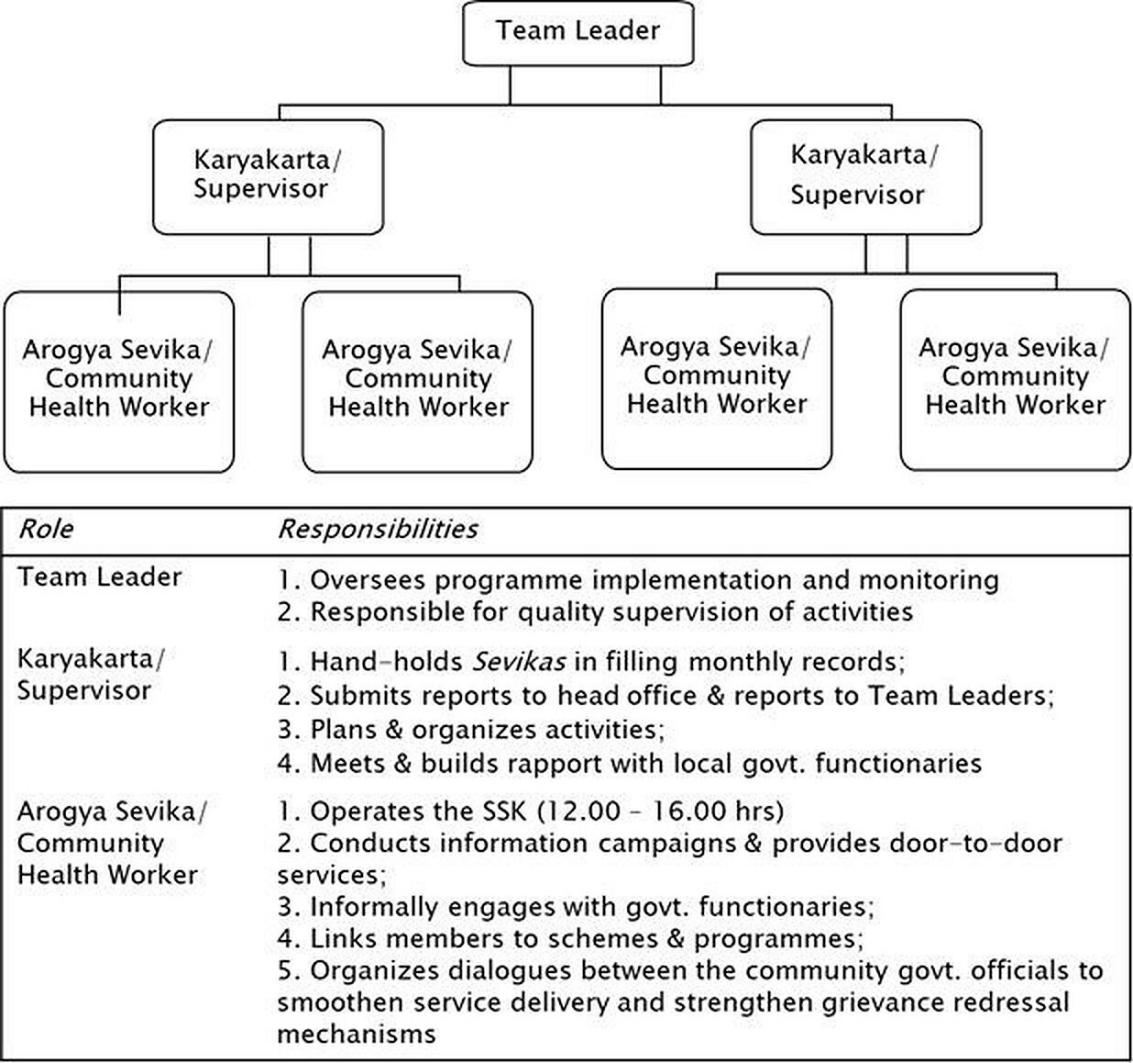
Human resource structure at the SSK. SSK, SEWA Shakti Kendras.

A team of two *sevikas* manages each SSK (figure 3). They are recruited, trained and paid a stipend. The *karyakarta* or supervisor provides hand-holding support in planning and organising activities, building rapport with local government functionaries and developing monthly activity reports. Team leaders (one each per urban/rural district) are responsible for overseeing programme implementation and monitoring activities in SSK areas.

### Implementation processes: community engagement channels

**Figure 4:**
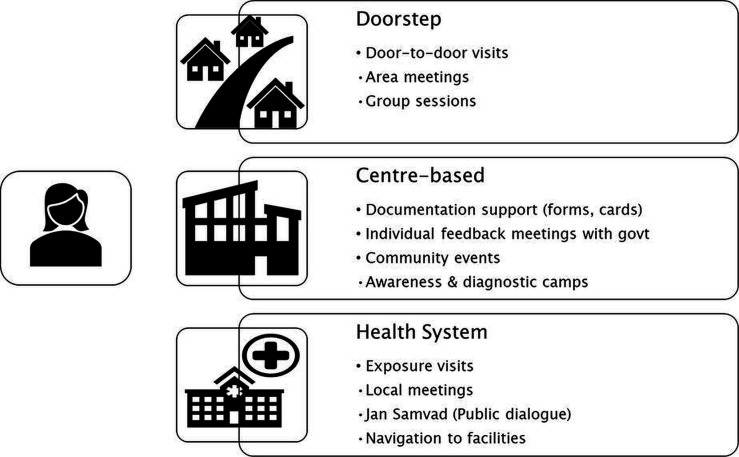
Community engagement channels.

SSKs used multiple community engagement channels to provide information, support and health system navigation, while building linkages with local government. *Sevikas* engaged with women through: (1)home visits and small-group meetings; (2)centre-based activities: one-to-one support for those who visit SSKs physically and local SSK-run community events with registration desks for identification documents and health check-ups and (3)exposure visits with the health system, for women to learn how to obtain documentation and use insurance cards (figure 4). SSKs also conducted informal meetings with local officials and broader, public dialogue meetings with the local government.

Between January 2020 and March 2021, workers across 6 SSKs acheived 12,730 contacts with households through individual visits, community meetings and events (table 2). *Sevikas* implemented four components of the SSK intervention: information provision; documentation support; health system navigation and government linkages, as described below. SSK teams report spending approximately 40%–50% of their time on health insurance, although this varied by SSK.

**Table 2:** Total contacts by SSK across intervention channels

SSK area	Total households reached (January 2020–March 2021)
Danilimda	1928
Makubhai na chapara	2204
Thorithambha	1763
Ghoda	2439
Varna	2155
Chaloda	2241
Total	12 730

SSK, SEWA Shakti Kendras.

#### Information provision

Many women reported attending local meetings, community events or health camps organised by the SSKs. They described gaining information on government schemes, including PMJAY and health and nutrition in events conducted by the SSK.

Since Ramilaben [*sevika*] conducts meetings here, we get information on government benefits and how they can help us. This is when we come to know this information; otherwise, we didn’t know anything. Urban member, 41 years

Once Sumanben [*sevika*] tells us, then we come to know about services and schemes. If she doesn’t come to our area, then we don’t know. Sumanben brings the correct information, and then ensures our work is done. Rural member, 45 years

I learned about the important places where things get done. I’m an educated woman, but women from my community typically don’t go to school. We were told that we don’t need to pay five hundred or thousand rupees (US$1=75 Indian Rupees, April 2020) to anyone/an agent to obtain an identity card. You can go to SEWA, get the form there and get help. Rural member, 30 years

Some respondents reported that *sevikas* visited them at home and gave them information on PMJAY and other government schemes. Women who found it difficult to step away from work or household commitments particularly appreciated this doorstep approach.

She comes to tell me to attend meetings, but there is no one in my home to do the housework if I leave. So [attending meetings] doesn’t suit me. But she comes home to give me information, even though other women physically go to meetings with her. Rural member, 58 years

#### Documentation support

PMJAY is an entitlement-based scheme based on vulnerability criteria, and thus has no formal enrolment process. It sends eligibility letters and generates cards for covered households; beneficiaries show the card when seeking covered health services. *Sevikas* provided hand-holding to navigate these processes, particularly advice on obtaining poverty-status documentation, such as the below poverty line card. Some women who had received a letter confirming their eligibility for PMJAY were unclear about the purpose of the scheme until a *sevika* explained the benefits. Respondents described how the *sevika* accompanied them to government offices to obtain documentation and assisted in submitting forms. In some instances, she followed-up with government offices on the status of cards.

Since Rajiben [*sevika*] keeps everything on track, we got the card. Otherwise, I would have had to go on my own. The next day, she came to ask whether I have got the card. If not, she would go to check. Rural member, 58 years

If someone wants to make a health insurance card, ration card or income certificate then the *sevika,* Seemaben goes with them and gets these cards done for them. She even came along with me to the offices. Urban member, 54 years

She [*sevika*] takes us to the *Mamladar* [local administrator] office and introduced us to all the officers. Rural member, 45 years

They took us to the place where Aadhar [identity] cards are issued; and then took us to learn about [health insurance] cards. All of the officials treated us very well. Urban member, 41 years

#### Exposure visits

Respondents reported participating in exposure visits to local health centres, hospitals and government offices. *Sevikas* took small groups of women to local health centres and government offices, either by foot or shared transport, with costs shared by women and supported by LSST if needed. Women reported that only the men of the family typically would meet government staff. Even when women went, they were accompanied by men and did not have the chance to interact directly with officials. However, during these exposure visits with *sevikas*, women asked questions directly to understand their entitlements. SSK exposure visits built women’s confidence in navigating the health system. They made otherwise unfamiliar spaces accessible.

Initially, we feared that if we go there then no one is going to touch us or give us medicine or speak properly with us. There was fear, so no one used to go there. But now we learned there is no need to be scared. They [government officials] talk peacefully, listen to us carefully, they help if there is any problem. So, we are going there [to these facilities] now. Urban member, 48 years

While *sevikas* viewed their role as a link to services, they emphasised it was not simply a ‘hand-holding’ mechanism or navigation service; SSKs empowered women, intentionally and actively.

You cannot keep holding a child’s hand to walk; we give our sisters [SSK beneficiaries] experience through the exposure visits to eventually use services themselves. Awareness is only so much; they need to *see* the offices and meet officers to build their confidence. Rural *sevika*

For many respondents who availed of PMJAY, a *sevika* recommended an empanelled hospital. Once in the hosptials, most respondents managed themselves or with help from family. In some instances *sevikas* stayed through the admission process.

Sairaben and Lalitaben [*sevikas*] informed me that the local government hospital is good and with the health insurance card, treatment will be free of cost. … They came with me as well, and later I was also able to go alone. Urban member, 42 years

She came with us wherever we needed to go at first, then she was there over phone to help us. She left after my mother-in-law was admitted to the hospital. … She helped us in getting the documents ready and she waited until the process was done and bed was provided. Rural member, 30 years

Yes, Ramilaben [*sevika*] gave the information [about the empanelled hospitals] where the insurance card can be used. She advised me [to] enquire if they can waive off the medicine cost. Urban member, 41 years old

### Contextual factors

#### Rural–urban differences

SSK staff highlighted how centres operated differently across contexts. In urban areas, women were generally able to identify appropriate services for their needs, but needed support in using technology and navigating facilities. In rural areas, SSKs focused on awareness about empanelled hospitals and navigation support across government offices. Large community events, often held in partnership with government facilities, were successful in rural areas. Hundreds of people would attend camps that facilitated linkages to treatment, including hospital treatment, through primary healthcare screening and services. However, urban SSKs relied more on home visits and group meetings, primarily due to space constraints.

An LSST staff member noted: We had to keep adapting the model based on the local situation and women’s needs. Most urban women know about hospitals but need support with the procedures or identification documents.

#### Government partnerships

LSST staff identified SSKs’ consistent and close coordination with local government particularly ASHAs (community health worker) and medical officers, as their primray strength. Linkages made information provision a useful activity; otherwise, as a *sevika* noted: ‘what is the purpose of telling people about schemes without being able to help get the service.’ SSK staff highlighted how the centres facilitated community engagement with a range of government schemes. A *sevika* articulated: ‘We are the bridge. We know women and their families on one side, and we know the government system on the other: we are in the middle to help.’

Government engagement was continuous, through multiple informal and formal channels. These include *Jan Samvad* meetings between communities and government officials to learn about new schemes and community engagement bodies constituted by the government (eg *Rogi Kalyan Samitis*, VHSNCs, *Mahila Arogya Samitis*) and to address grievances. Informal meetings with local government officials, doctors and other staff at health facilities, especially community health workers, supported coordination and specific follow-up. *Sevikas* reported regularly updating officials on progress or issues faced by community members and partnering on outreach for government programmes.

We hold a meeting with the local government officials at least monthly, where we share our work with SEWA and also let them know what our members need. We ensure that these meetings look for a solution and are not just negative. Rural *sevika*

SSK staff noted that women in their areas had not felt the need to use the PMJAY telephone grievance mechanism regularly. Instead, women came to them directly to help solve procedural issues with benefits, either directly to the centre or through home visits and community meetings. SSK health supervisors emphasised the importance of working in partnership with front-line workers to link women to the public health system. In addition, *sevikas* identified the need to strengthen and expand existing mechanisms for community participation in health, such as ASHA to engage directly with health insurance.

The VHSNCs [village health, sanitation and nutrition committee] need to be aware of health insurance too—so there is a link between them and Ayushman [PMJAY]. Rural *sevika*

The ASHA should be a better source for health insurance too, but she needs support to follow through to ensure services reach. They do excellent work on health, but she can’t monitor or follow-through the way we can, as we have to respond to our members. Rural LSST supervisor

Government counterparts, recognising the benefit of SSKs, facilitated exposure visits and processes. LSST staff reported that the willingness to partner with community-based organisations varied by individual officers. SSKs in areas with longstanding linkages to ASHAs and community health processes were able to develop stronger systems for outreach and resolve members’ concerns. Newer SSKs required time to develop these relationships, although SEWA’s pre-existing work in the area expedited this process.

Where there was no SSK before, we had to meet government officers weekly, to get to know them. In areas we’ve been working for a long time, the ASHAs and government already know us. Rural *sevika*

In areas where SSKs functioned well, with strong government linkages, the lack of empanelled hospitals close to members in rural areas was a persistent barrier to using PMJAY. *Sevikas* noted that women commonly came to them for informal quality assessments of hospitals or advice on where to seek services close to home. Some reported that information on empanelled hospitals was not sufficiently updated online, although this may have been a temporary disruption during COVID-19.

Outside of SSKs, some respondents received information on health insurance at facilities. Typically, doctors would recommend using the scheme if respondents were eligible and lacked the financial means to pay. Respondents found hospital staff and PMJAY information desks helpful in navigating health facilities. Some reached hospitals without any information about health insurance accepted at the hospital, but learned there. They also recalled information boards inside the hospital on health insurance schemes, as well as direct follow-up by the government.

When we went to the hospital, they already had all the posters put up of [the insurance schemes]. We showed our poverty identification card to the doctor. Then the doctor asked us to go to two-three offices, where we showed the card and could use insurance. Urban member, 40 years

I get calls from Ayushman Bharat, they ask me if I’m facing any issues, or if there’s been any problem with the treatment. They even ask if the hospitals asked me for money. They inquire and follow up over the phone. Rural member, (age not available)

#### Literacy and digital literacy

Literate or digitally-savvy respondents could look up information independently on their phones or understand information boards, while women who were not literate depended on SSKs.

I got the information from my phone. I took my Aadhar card, voter ID card and then generated the insurance [MA] card. Rural member, 54 years

My son searched a lot about the PMJAY card on Google. A lot of information was there, including a helpline number. Rural member, 58 years

For women who are not literate, the SSK meetings tell them important things, or they get this information from the SSK. Rural *sevika*

I’m educated and so is my husband and brother-in-law, so we would go to the offices independently to solve any problems. Rural member, 30 years

#### Trust in SEWA

Women highlighted the role of the local, trusted SEWA worker in helping access government schemes, particularly PMJAY. Nearly all respondents knew of their local *sevika*, if not the SSK itself. Respondents perceived her to be a credible source of information on government schemes, on health and other services. They trusted her, felt she was approachable and reliable for any help they required, at any time:

Whatever help women require, they [*sevika*] find a way to help. Rural member, 45 years

If there is any government work, she makes sure she can help: taking patients to the health centre, helping in the ration card—she does everything. Rural member, 58 years

Sumanben [*sevika*] had almost 30–35 years of experience. That’s why I have faith in her and other women also have faith in her. That’s why whenever we have any work or need help, we tell her. She helped us access many other services. Through her, we have all the information we need. Rural member, 45 years

There are many benefits to the SSK. Even if we have any work at midnight, then [also] Ramolaben [*sevika*] comes. If we need anything, she comes immediately. Urban member, (age not available)

## Discussion

The SSK model aimed to improve women’s capacity to access health insurance through trusted *sevikas,* who provided awareness, health system navigation support and facilitated government linkages. LSST envisaged SSKs as a ‘one-stop solution’ for community members, working across government departments and a range of schemes to ensure services are available at women’s doorsteps. Waddington et al have highlighted three elements of effective community engagement interventions: strong local buy-in with participatory processes; culturally specific interventions that address local barriers to inclusion of vulnerable groups; and participatory planning processes that support local capacity for collective action.^1^ The SSK model was locally rooted and responsive, with interventions that engage women and vulnerable groups through flexible approaches that varied across areas. However, there was limited collective action to improve the implementation of the health insurance scheme Instead, *sevikas* built on LSST’s credibility and existing linkages with the government to negotiate individual cases.

SSKs are somewhat in the middle of the spectrum of accountability models for health insurance, ranging from legally mandated accountability to privatised service navigation support. In Colombia, legal directives require insurers to establish formal user associations to monitor quality of care and address grievances, whereas in Indonesia volunteer groups engage directly with insurers as activists. In both India and Ghana, social entrepreneurs and other private actors serve as agents who help individuals use health insurance for a fee.^4 5 27^ SSKs, not formally mandated, work closely with both government and community members, but do not play a formal, paid-agent role.

Our findings highlighted three elements of the SSK model that facilitated effective implementation: (1) local leadership (2) government partnerships and (3) a flexible approach to meet a range of needs. SSKs are run by *sevikas* who are SEWA members themselves; they are local, trusted and understand the dynamics of their communities. SSKs engaged with government at multiple levels, including: ASHAs, *panchayati raj* institutions; local administrative offices to obtain documentation and with health services. Rather than engage in a monitoring role, SSKs prioritised working as a ‘bridge’.

SSK workers’ familiarity with services beyond health insurance emerged as an important enabler. The ability to respond to multiple needs and challenges, rather than a single agenda, built trust among communities. SSKs bridged different gaps such as digital literacy, lack of social networks, or poor outreach from the health system amongst members, Types of support thus varied across individuals and urban–rural settings.

The model operated on two underlying principles: (1) trust, both in *sevikas* and SEWA and (2) an empowering approach to build women’s independent capacity to use health insurance. Women trusted health workers for their local knowledge, constant availability and their ability to work across government systems. SSKs drew on SEWA’s history as a women’s organisation to respond to women’s needs in a localised, organising approach to access entitlements.^23^

We characterise the model as empowering to underscore its difference from government-mandated associations or agent-based models. Rather than simply provide a service, SSKs engaged in different ways to strengthen women’s confidence and capabilities. The approach helped women independently seek services and negotiate government systems, thereby addressing some gender-based barriers to health insurance utilisation. Previous experience indicated that relying only on one-way information transfer, even through local health workers, was not sufficient to influence use of health services. Rather, the active participation of members and *sevikas*’ facilitation was critical.^28^ SSK exposure visits were, as articulated by members and *sevikas* alike, an important tool for women to ultimately seek services themselves, rather than relying on navigation support. Group meetings held by *sevikas* facilitated sharing experiences and identifying solutions.

These enabling factors, however, also present challenges to implementation in new areas. While several features of the model are similar to community engagement initiatives globally, specific contextual features of LSST in Gujarat facilitated implementation. Most importantly, strong government partnerships took time to build. SEWA’s long-standing presence and reputation was likely an important influence on government responsiveness SSKs also relied on individual *sevikas*’ ability to craft activities based on women’s needs. Accordingly, *sevikas* themselves required a strong system of support, provided by LSST.

### Transferability

In India, there are at present limited formal opportunities for community engagement within PMJAY, beyond in-built accountability mechanisms such as a mobile app for beneficiaries and a grievance redressal hotline. Other potential government platforms or pilots include Government-run Common Service Centres (which vary by state) that provide computerised service delivery for several schemes and access to digital services, including telehealth consultations. Some state health insurance programmes have experimented with working more closely with ASHAs to identify eligible families, provide information, mobilise beneficiaries to create cards and referrals to public hospitals.^29^ A technology-based pilot, *Haq Darshak*, has trained self-help group members as agents who offer fee-based enrolment in welfare schemes, to empower individual women to earn an income while capitalising on group networks.^30^

Greater community engagement within PMJAY could draw from the experience of India’s National Health Mission, which introduced community action for health in 2005 through decentralised planning through VHSNCs, community-based monitoring and feedback, awareness-raising through front-line workers and public hearings to increase accountability and improve services.^31^ Despite mixed success nationally, states learnt that partnering with local organisations enabled inclusion of marginalised community members and capacity building.^32^ Recently proposed mechanisms for greater community engagement with PMJAY include involving these established platforms, as well as community health workers, women’s groups and patients’ groups, through social audits, community feedback and public dialogue meetings.^33^ Additional efforts proposed, though not yet piloted, to support bidirectional referral linkages between health insurance and HWCs include facilitation by Ayushman Bharat Counselling Centres to support informed choices for treatment, referrals for follow-up care and grievance redressal. 34

As India’s health insurance schemes continue to experiment with improving outreach and utilisation, particularly among women, the SSK experience offers important lessons. Local leadership, community-based, one-stop health system navigation and partnerships between community-based organisations and government are key. Scaling up community engagement will require integrating these principles to empower existing structures such as ASHAs and VHSNCs to engage with health insurance—while also working with local community organisations and women’s groups--to improve women’s use of insurance.

### Strengths and limitations

This study aimed to contribute to the limited literature on implementation of community engagement initiatives specific to publicly funded health insurance. Most experience with accountability and engagement-focused initiatives in India examines community-based health insurance schemes.^35^ Local trust in LSST allowed for phone interviews with ease, but COVID-19 restrictions prevented observations and in-person interviews with government officials, as originally planned. The study was conducted in equal partnership between LSST and the research team. Accordingly, we prioritised findings that can contribute to practice, while expanding the implementation research literature on health insurance. Future research that builds on the pathways identified in this study will be important to examine the effectiveness of and variation across SSKs, alongside analyses of the level, necessity and quality of care provided at empanelled hospitals.

## Conclusion

India has a long history of encouraging community involvement in the health system, spanning village-level health committees, community-based monitoring of services and mobilisation through participatory learning and action facilitated by health workers.^36–38^ As expected, these efforts vary widely in their intensity of engagement and the influence of contextual factors.^2 39^ The SSK model demonstrates how community engagement can be implemented through a local, flexible and community-run model that is a literal ‘bridge’ between people and public services, including health insurance. Localised partnerships between community-based organisations and governments may be the first step towards widescale community engagement that underpins moving closer to UHC.

## Data Availability

Data are available on reasonable request. Data are available on reasonable request, with data sharing agreements. Data include pseudonyms/de-identified interview transcripts. Data requests can be sent to SD and ST.
