# Supplementary material for: ‘We are the bridge’: an implementation research study of SEWA Shakti Kendras to improve community engagement in publicly funded health insurance in Gujarat, India
Source: BMJ Glob Health. 2022 Sep 23;7(Suppl 6):e008888. doi: 10.1136/bmjgh-2022-008888 (PMC9511541; doi:10.1136/bmjgh-2022-008888)
Supplement: Supplementary data [file bmjgh-2022-008888supp001.pdf]

SUPPLEMENTAL MATERIAL

Study Sites and Population (Ahmedabad district)

Area (rural block or urban ward)	Population
Rural (Block, Village)	
Viramgam (Thorithambha)	2887
Viramgam (Ghoda)	3739
Dholka (Varna)	4239
Dholka (Chaloda)	8641
Urban (City, ward/area)	
Ahmedabad (Danilimda)	5250
Ahmedabad (Makubhai na chapara)	3000
**Total**	**27,756**
